# The Optimization of an Anti-VEGF Therapeutic Regimen for Neovascular Glaucoma

**DOI:** 10.3389/fmed.2021.766032

**Published:** 2022-01-10

**Authors:** Ling Bai, Yanfen Wang, Xindi Liu, Yuping Zheng, Wenjing Wang, Na He, Farheen Tariq, Feng Wang, Shu Zhang

**Affiliations:** ^1^Department of Ophthalmology, The Second Affiliated Hospital of Xi'an Jiaotong University, Xi'an, China; ^2^Department of Clinical Medicine, Zhejiang University School of Medicine, Hangzhou, China; ^3^Experimental Teaching Center for Clinical Skills, The Second Affiliated Hospital of Xi'an Jiaotong University, Xi'an, China; ^4^Department of Geriatric Medicine, The Second Affiliated Hospital of Xi'an Jiaotong University, Xi'an, China

**Keywords:** neovascular glaucoma, pan-retinal photocoagulation, anti-VEGF therapy, anti-glaucoma surgery, glaucoma

## Abstract

This study investigates the safety and efficacy of conbercept injection through different routes for neovascular glaucoma (NVG) treatment, in which seventy-four patients (81 eyes) with NVG caused by ischemia retinopathy had participated. Patients were divided into three stages according to the progression of NVG and were randomly assigned to receive intracameral or intravitreal conbercept injection. After conbercept injection, patients experienced improved best-corrected visual acuity (BCVA), good intraocular pressure (IOP) control, and neovascularization of Iris (NVI) regression. In stage III, patients required trabeculectomy with mitomycin C plus pan-retinal photocoagulation (PRP) to achieve complete NVI regression. Compared to the intravitreal group, the intracameral group had significantly lower IOP in 2 days in stage III and 1 day in stages I and II after injection, complete NVI regression before PRP in stages I and II, and better NVI regression in stage III. The rates of hyphema after trabeculectomy and malfunction filtering bleb suffering needle bleb revision were lower in the intracameral group, but only the hyphema rate was significantly different. Injections through different routes are all safe. We recommend intravitreal injections for patients in stages I and II, but for stage III, intracameral injection is better, and trabeculectomy with mitomycin C should be conducted within 2 days after injection to maximally reduce the risk of perioperative hyphema.

**Trial Registration:** ClinicalTrials.gov, identifier NCT03154892.

## Introduction

Neovascular glaucoma (NVG) is a refractory type of secondary glaucoma and often leads to frustrating treatment and blindness. Conventional anti-glaucoma medicines cannot effectively reduce intraocular pressure (IOP) in NVG, and filtering surgery of anti-glaucoma often fails due to severe inflammation and perioperative hyphema. Cyclodestructive surgery, such as cyclophotocoagulation (CPC) and cyclocryotherapy, can decrease IOP. However, without precise quantification, it often leads to hypotony and phthisis (1).

The progression of NVG can be divided into three stages: I. Rubeosis iridis: Neo-vessel can be seen at the iris (NVI) or anterior chamber angle (NVA). Patients have normal IOP. II. Open-angle glaucoma: Fibrovascular tissue grows over the trabecular meshwork and obstructs aqueous outflow. Patients have increased IOP, but the angle is open in various regions. III. Closed-angle glaucoma: Contraction of fibrovascular membrane produces secondary synechia of the trabecular meshwork and peripheral iris. The angle is closed. Patients suffer from severe pain, abruptly high IOP, and often disastrous vision loss (2).

The common underlying initiating mechanism for NVG is ocular ischemia, which is often secondary to ischemic central retinal vein occlusion (ICRVO), proliferative diabetic retinopathy (PDR), and ocular ischemic syndrome (OIS). Neovascularization is the result of the imbalance between pro- and anti-angiogenic factors. Vascular endothelial growth factor (VEGF) is one of the most important pro-angiogenic factors, and its expression is enhanced from the ischemic retina. Previous literature has described that VEGF levels are significantly higher in aqueous humor and vitreous patients with NVG (3–5).

Currently available anti-VEGF agents are composed of monoclonal IgG antibodies and recombinant fusion proteins. Ranibizumab (Lucentis) and Bevacizumab (Avastin) represent monoclonal IgG antibodies and bind to all VEGF-A isoforms. The recombinant fusion proteins are a new generation of anti-VEGF agents that can block not only all VEGF-A isoforms but also VEGF-B and placenta growth factor (PlGF). The representatives are aflibercept and conbercept. They are similar in structure, consisting of the second Ig domain of VEGFR1 and the third Ig domain of VEGFR2 fused to the Fc portion of human IgG1. Conbercept also has an additional fourth Ig domain of VEGFR2, which stabilizes receptor dimers and locks VEGF to the receptor in a rigid manner, which may have a much higher affinity to VEGF (6). Until now, all four of these medicines have been used in neovascular age-related macular degeneration (wet-AMD, wAMD), and another ocular disease with VEGF that plays an essential role in Its development), and conbercept has demonstrated superiority over the others (7). However, regarding NVG, there is not yet a clinical report about conbercept.

This study aimed to evaluate the safety and efficacy of conbercept injection through different routes for NVG treatment and to optimize the anti-VEGF therapeutic regimen for NVG.

## Methods

### Study Design

This single-center study was designed as a prospective, comparative effectiveness study to assess the safety and efficacy of conbercept through different injection routes in patients with NVG. The Medical Ethics Board of the Second Affiliated Hospital of Xi'an Jiaotong University has approved this study. It complied with the Declaration of Helsinki and was registered at clinicaltrials.gov (NCT03154892). Informed consent was signed from all patients before study enrollment.

### Patients

Eighty-five patients (90 eyes) were recruited for this study in the ophthalmology department of the Second Affiliated Hospital, Xi'an Jiaotong University. Seventy-four patients (81 eyes) completed at least 6 months of follow-up. The information of the participants is presented in Table 1. We included the patients with NVG whose causes were ischemia retinopathy, such as PDR and ICRVO. Carotid occlusive disease (such as OIS) causing NVG was excluded because these patients require vascular surgical treatment. Patients who had received intraocular administration of anti-VEGF drugs within 60 days in the study of eye or systemic administration within 180 days before Day 1 and patients with any contraindication for anti-VEGF agents were excluded.

**Table 1:** Demographic data for 81 eyes in this study.

**Characteristic**	**Patients (***n*** = 74)**	**Eyes (***n*** = 81)**
Age(y), mean(range)	64.2 (39-79)	66.8 (39-79)
Male sex, *n* (%)	51 (68.9)	56 (69.1)
**Cause of neovascularization**		
PDR, *n* (%)	43 (58.1)	50 (61.7)
CRVO, *n* (%)	31 (41.9)	31 (38.3)

### Ophthalmic Examination

At the initial time, all participants of this study went through the following set of tests: best-corrected visual acuity (BCVA) measured with Snellen chart, IOP by Goldmann applanation tonometer, slit lamp examination for the anterior segment, gonioscopy, detailed fundus examination, and fundus fluorescein angiography (FFA) if the cornea was clear.

After evaluation, patients were divided into three stages according to the progression of NVG. Eligible patients were randomly assigned in a 1:1 ratio to receive either intracameral or intravitreal conbercept injection. Randomization was performed centrally by using a computer-generated random-number sequence and stratified according to the stages (Table 2).

**Table 2:** The initial statement before conbercept injection.

**NVG stages**	**Eyes (***n***)**		**Mean initial IOP**	**Mean BCVA**	**NVI**			**NVA (/4)**				**PAS (/4)**			
			**(mmHg)**	**(Log MAR)**	**+**	**++**	**+++**	**1**	**2**	**3**	**4**	**1**	**2**	**3**	**4**
I	Intracameral	10	17.1 ± 2.7	0.37 ± 0.15	20	0	0	7	0	0	0	0	0	0	0
	Intravitreal	10													
II	Intracameral	10	28.2 ± 2.9	0.61 ± 0.19	4	13	3	4	12	4	0	0	0	0	0
	Intravitreal	10													
III	Intracameral	21	43.1 ± 3.6	1.81 ± 0.62	0	2	39	0	0	5	14	0	0	3	16
	Intravitreal	20													

*At stage III, because of the corneal edema or severe pain, gonioscopy could not be conducted in 22 eyes.

*IOP, Intraocular pressure; BCVA, Best-corrected visual acuity; NVI, Neovascularization of iris; NVA, Neovascularization of angle; PAS, Peripheral anterior synechia; 1/4, 2/4, 3/4, 4/4, Quadrants of the anterior chamber angle with NVA or PAS*.

### Intracameral Conbercept Injection

Under topical anesthesia with 0.5% proparacaine hydrochloride eye drops, the 0.5 mg/0.05 ml injection of conbercept (Chengdu Kanghong Biotech Ltd, Sichuan, China) in the anterior chamber at the cornea limbus was performed using a 30-gauge needle. This procedure was assisted by a surgical microscope and did not need extra anterior chamber paracentesis.

### Intravitreal Conbercept Injection

Under topical anesthesia as described above, injection conbercept 0.5 mg/0.05 ml was performed at 4.0 mm posterior to the limbus in phakic eyes or 3.5 mm posterior to the limbus in aphakic or pseudophakic eyes through a 30-gauge needle. Anterior chamber paracentesis was necessary if marked IOP elevation was observed at the end of intravitreal injection in stage III.

### Pan-Retinal Photocoagulation

Pan-retinal photocoagulation was conducted under a slit lamp using a LIGHTMED 577 wavelength fundus laser machine. The spot size was 300 μm, and the level II–level III reactions were appropriate for photocoagulation of the retina. PRP was usually completed within 1 week. The setting for total photocoagulation was 1,800–2,400 spots. At the time of follow-up, supplemental photocoagulation based on FFA was necessary in case of NVI recurrence.

For patients with refractive media opacity, the RPR could not be conducted. We first performed the surgery to treat the cloudy media in these cases, such as phacoemulsification for cataract, anti-glaucoma surgery for corneal edema, and pars plana vitrectomy with endolaser photocoagulation for vitreous hemorrhage.

### Anti-glaucoma Surgery

Anti-glaucoma surgery was indicated in the case of failed IOP control despite having maximal tolerated topical hypotensive medications or patients who cannot receive PRP due to corneal edema. The surgery used in this study was trabeculectomy with mitomycin C as previously described (8, 9).

### Follow-Up

All the patients were observed 1 day, 2 days, and 1 week after conbercept injection. If the patients received trabeculectomy, they were observed on 1 day and 1 week after surgery. They were then followed up every month for at least 6 months after PRP. BCVA, IOP, the presence of NVI, and the complications after trabeculectomy were documented at each visit.

### Statistical Analysis

SPSS version 23 (SPSS Inc., Chicago, IL, USA) statistical software was utilized to analyze the data. Student's *t*-test was used to calculate the differences between two groups in IOP and visual acuity before and after treatment. In contrast, the comparison of NVI regression rate and incidence of complications were calculated using Fisher's exact test, the Pearson chi-squared test, or the continuity correction chi-squared test according to the sample size. Tests were considered significant at *p* < 0.05.

## Results

### Visual Acuity

As listed in Table 3, BCVA is improved in 51 (63%) eyes, while it remains unchanged in 30 (37%) eyes. Regarding eyes, in stage I and stage II, BCVA after treatment was not statistically significantly improved compared to the initial statement in either the intracameral group or the intravitreal group. However, the BCVA in stage III was all significantly improved after treatment. The difference between the intracameral and intravitreal groups was not significant. Interestingly, 2 eyes with light perception (LP) at the initial statement had achieved improvements in visual acuity of 0.02 and 0.04 after the whole course of treatment.

**Table 3:** The best-correct visual acuity (Log MAR) before and after treatment.

**Stage**	**Intracameral injection group**			**Intravitreal injection group**		
	**Before treatment (Initial statement)**	**After treatment**	***P***	**Before treatment (Initial statement)**	**After treatment**	***P***
I (20eyes)	0.35 ± 0.15	0.29 ± 0.12	*t* = 0.91 *P* = 0.37	0.39 ± 0.15	0.34 ± 0.10	*t* = 0.87 *P* = 0.4
II (20 eyes)	0.63 ± 0.22	0.49 ± 0.14	*t* = 1.66 *P* = 0.11	0.59 ± 0.18	0.47 ± 0.09	*t* = 1.86 *P* = 0.08
III (41 eyes)	1.82 ± 0.67	1.12 ± 0.33	*t* = 4.32 *P* <0.001	1.80 ± 0.59	1.17 ± 0.32	*t* = 4.15 *P* <0.001

### Intraocular Pressure

**Figure 1:**
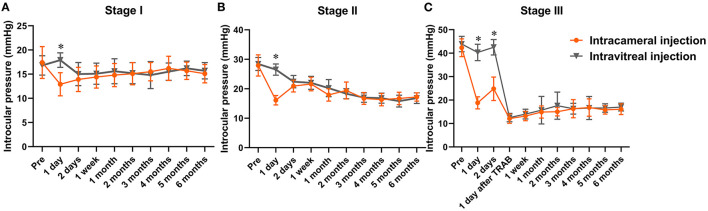
Intraocular pressure profile following conbercept intracameral/intravitreal injection for NVG. *Indicates *p* < 0.05. NVG, neovascular glaucoma.

For stage I, in the intracameral injection group, the IOP before treatment was 17.4 ± 3.3 mmHg, which was decreased to 12.9 ± 2.4 mmHg on the first day after injection, then slightly increased to 13.9 ± 2.5 mmHg on the second day and remained stable for the follow-up period. In the intravitreal injection group, the IOP before treatment was 16.8 ± 2.0 mmHg, slightly increased to 17.9 ± 1.5 mmHg on the first day after injection, which was significantly different from the intracameral injection group (*p* < 0.01). On the second day, IOP was decreased to 15.0 ± 2.4 mmHg and remained stable for the follow-up period (Figure 1A).

For stage II, the pre-treatment IOP was 27.9 ± 3.6 mmHg in the intracameral group and 28.4 ± 2.2 mmHg in the intravitreal group. On the first day after conbercept injection, the IOP was decreased to 16.1 ± 1.6 mmHg in the intracameral group and 26.5 ± 1.9 mmHg in the intravitreal group. The difference in IOP between the two groups was statistically significant (*p* < 0.001). On the second day, the IOP was increased to 20.9 ± 2.0 mmHg in the intracameral group and decreased to 22.4 ± 2.1 mmHg in the intravitreal group. At this time, we applied topical hypotensive drugs for eyes with IOP > 21 mmHg and completed PRP within 1 week. Through the whole follow-up period, 18 eyes achieved good IOP control, and 1 eye in the intracameral group needed administration of two topical hypotensive drugs. Even with maximal-tolerated topical hypotensive drugs, 1 eye in the intravitreal group at 1 month did not achieve good IOP control until trabeculectomy with mitomycin C was performed (Figure 1B).

For stage III, the pre-treatment IOP was 42.3 ± 3.8 mmHg in the intracameral group and 43.9 ± 3.3 mmHg in the intravitreal group. On the first day after conbercept injection, the IOP was decreased to 18.8 ± 2.6 in the intracameral group and 40.3 ± 3.5 mmHg in the intravitreal group. The difference in IOP between the groups was significant. On the second day, the IOP was increased to 24.8 ± 5.0 mmHg in the intracameral group and remained 42.5 ± 3.3 mmHg in the intravitreal group. Because the IOP tended to increase in the intracameral group and was maintained at a high level in the intravitreal group, we performed trabeculectomy with mitomycin C on this day for all patients. One week later, we started PRP. Because of retinal hemorrhage, 1 eye in the intracameral group could not complete PRP at 1 month. We repeated conbercept intravitreal injection and waited for the hemorrhage to be absorbed to complete PRP. For the four eyes not suitable for PRP due to cataract and/or vitreous hemorrhage, we first performed phacoemulsification and/or vitrectomy and endolaser and then completed PRP. At the end of the follow-up time, the IOP was controlled well in the two groups. Two eyes needed the administration of two topical hypotensive drugs (Figure 1C).

### NVI Regression

For stage I, all the NVI was regressed 1 day after conbercept injection. We then started PRP, and there was no recurrence of NVI. For stage II, all the NVI was regressed 1 day after injection in the intracameral group, but in the intravitreal group, 5 eyes had remaining NVI 1 day after injection, and 2 eyes were slightly persisting until 1 week later. After PRP completion, the remaining NVI had disappeared at 1 month.

For stage III, 17 eyes (81%) had complete NVI regression 1 day after intracameral injection and did not recur on the second day. Only 8 eyes (40%) had complete NVI regression in the intravitreal group, and residual NVI was present in 12 eyes (60%) until the second day after injection.

Due to the failure of IOP control in the intravitreal group, all the eyes in stage III had trabeculectomy with mitomycin C on the second day after conbercept injection to avoid the continued damage from high IOP to the optic nerve in this study. Excitingly, the residual NVI of the four eyes in the intracameral group disappeared on the first day after trabeculectomy. The regression rate of NVI in the intravitreal group was increased to 60%, and the IOP was decreased 1 week after trabeculectomy. We completed PRP, and all the NVI had regressed 1 month later. Two eyes in the intracameral group and three eyes in the intravitreal group had a recurrence of NVI at the 3- and 4-months visits, respectively, and extra-PRP was promptly performed. We did not find any NVI recurrence at the end of the follow-up (Table 4).

**Table 4:** Regression rate (%) of NVI following conbercept injection.

			**Time after Injection**									
**Stage**	**Injection route**	**Initial**	**1 day**	**2 days**	**3 days (1 day after trab)**	**1 week**	**1mo**	**2mo**	**3mo**	**4mo**	**4mo**	**6mo**
I	Intracameral	0	100	100	100	100	100	100	100	100	100	100
	Intravitreal	0	100	100	100	100	100	100	100	100	100	100
II	Intracameral	0	100	100	100	100	100	100	100	100	100	100
	Intravitreal	0	50	60	60	80	100	100	100	100	100	100
III	Intracameral	0	81	81	100	100	100	100	90.5	100	100	100
	Intravitreal	0	40	40	50	60	100	100	100	85	100	100

*mo is for Month; NVI, neovascularization of Iris*.

### Complications

#### Hyphema

In stage III, hyphema occurred in three eyes (14.3%) in the intracameral group and 4 eyes (20%) in the intravitreal group in the process of conbercept injection. There was no significant difference between them (*p* = 0.94). Four of them were obvious and lead to a decrease in visual acuity the next day. We performed anterior chamber irrigation during trabeculectomy, and the visual acuity improved after treatment.

Twelve eyes in the intravitreal group still had NVI residue involving a larger area at the time of trabeculectomy than the intracameral group. Due to the NVI and failure of IOP control, hyphema occurred in five eyes (25%) after the surgery, which had been absorbed several days later. Tiny NVI residue only showed in four eyes in the intracameral group, and none of them experienced hyphema after trabeculectomy. These findings indicated that intracameral injection was a more effective route for stage III patients (*p* = 0.02).

#### Malfunction Filtering Bleb

At each follow-up time, the same experienced glaucoma expert evaluated the filtering bleb condition, and the needle bleb revision combined with 5-FU was conducted when a malfunction occurred (10). In total, five eyes (25%) in the intravitreal group and two eyes (9.5%) in the intracameral group required the procedure. Only a few patients needed needle bleb revision, with the difference between the two groups insignificant.

## Discussion

Pan-retinal photocoagulation is currently regarded as the gold standard treatment for NVG. By eliminating the ischemic retinal tissue, it permanently stops the stimulus for the production of pro-angiogenic factors and the subsequent neovascularization. However, PRP does not result in rapid regression of NVI/NVA, which usually takes several weeks to occur. During this period, patients suffer cumulative angle closure, elevated IOP, and eventually irreversible optic nerve damage. Based on these complications, some scholars have suggested anti-VEGF agents as substitutes for PRP in patients with NVG (11–13). The neovascularization process secondary to retinal ischemia can also be prevented by VEGF inhibitors (14). Several studies have reported better visual prognosis and IOP control following anti-VEGF injections in NVG (11, 15–17). However, because more than 10 times repetitiously injections are sometimes needed and the huge cost (13), this treatment method is not cost-effective and unaffordable in developing countries.

State Food and Drug Administration (FDA) in the People's Republic of China has approved the conbercept for the treatment of wAMD in 2013 (18). Still, the use of conbercept in treating NVG has not been reported. Because the anti-VEGF agent doses in the treatment of NVG and wAMD are similar to other VEGF inhibitors, we also referred to the treatment protocols of conbercept for wAMD (7). As we expected, the injection of conbercept could achieve increased visual acuity, controlled IOP, and NVI/NVA regression after 6 months of follow-up, which was consistent with reports using Bevacizumab for NVG (16, 19). These findings indicated that conbercept could be a more cost-effective candidate of anti-VEGF agent for NVG.

For the treatment regimen, we injected conbercept as the first step to reduce pro-angiogenic factors directly and rapidly and effectively alleviate disease progression, and we then completed PRP. During the therapeutic process, if cataract or vitreous hemorrhage disturbed PRP, we performed phacoemulsification or vitrectomy; if the high IOP caused corneal edema that disturbed the PRP, we first performed a trabeculectomy. Our study found that, after conbercept injection and prompt PRP, all NVG eyes in stage I and 95% of eyes in stage II had controlled IOP, prevented the damage progression of anterior chamber angle closure, and had no need for anti-glaucoma surgery. However, for the eyes in stage III, although the NVI/NVA could regress after conbercept injection, they required anti-glaucoma surgery, and completion of PRP was the goal.

Neovascular glaucoma is considered a frustrating cause for the failure of anti-glaucoma surgery. It is believed that perioperative hyphema and postoperative scarring are the common complications that relate to higher rates of trabeculectomy failure (20). For this reason, glaucoma drainage devices (e.g., Ahmed glaucoma valve) have been suggested to be the first choice for anti-glaucoma surgery in NVG. However, the use of anti-metabolites (mitomycin C) has improved the success rate of trabeculectomy (8). Similar success rates have been reported for trabeculectomy with mitomycin C compared to the Ahmed glaucoma valve in NVG (21). Trabeculectomy allows the aqueous humor to drain through a subconjunctival bleb to relieve the IOP, but a complete wound healing after surgery will constitute failure. The animal research from Seet et al. (22) showed that angiogenesis is an early response when a wound of filtering bleb is healing. A few clinical observations also confirmed that high VEGF expression and the failed surgery of glaucoma are significantly correlated (23, 24). These studies have suggested that anti-VEGF therapy is potentially useful in increasing the success of glaucoma filtration surgery. However, current literature has failed to ascertain the optimal route, timing of administration, and the full duration of effect.

In NVG treatment, VEGF inhibitors are usually injected through intravitreal routes (19, 25). It is believed that in patients with ischemic retinal diseases, VEGF is produced by a variety of cells, such as retinal pigment epithelial cells, Mueller cells, ganglion cells, retinal capillary pericytes, and endothelial cells (26). Chalam et al. (27) found that human ciliary epithelium was also an important source of the synthesis and secretion of VEGF in NVG, particularly, in eyes that were not responding to PRP. These patients needed repeated injection anti-VEGF drugs to resist VEGF synthesis from the non-pigmented ciliary epithelium. Bhagat et al. (16) reported that in terms of controlling IOP, the intracameral route was found to be most effective. Another study indicated that transient and sustained elevation in IOP, especially in stage III, might be due to the continuous intravitreal anti-VEGF injections (28). Intravitreal injection is easier and does not require the assistance of a surgical microscope. Although the IOP on the first day after injection was higher than with intracameral injection, it did not influence the effect. Thus, for NVG stages I and II, we suggest an intravitreal injection of conbercept. For stage III, the most important task is to create a safe time window for anti-glaucoma surgery. We found better IOP control and NVI regression in the intracameral group on the first day after injection in stage III. The possible explanation is that the agent can reach the iris and angle and act on the neovessels directly through the intracameral injection, and the local concentration of the anti-VEGF agent in the anterior chamber is higher than with intravitreal injection. However, due to the aqueous humor circulation and possible leakage after retracting the syringe, this advantage may soon start to diminish, as the IOP showed an upward trend on the second day. Although the time window is short, it provides us enough time to perform a trabeculectomy with less possibility of hyphema. Moreover, we did not notice any toxicity profile on the corneal endothelium and lens after intracameral injection that is why we recommend intracameral injection in advanced patients, especially in stage III.

With a combination of intravitreal ranibizumab and MMC, Kahook (29) observed more dispersed blebs with a lesser extent of vascularity in trabeculectomy, without using expensive glaucoma drainage devices. We also achieved good IOP control in patients who suffered trabeculectomy with mitomycin C combined with conbercept injection.

Hyphema is a risk factor, and it can cause the failure of trabeculectomy in NVG. A retrospective study showed that the association between preoperative intravitreal injection bevacizumab and postoperative hyphema was insignificant (20). This agrees with our results that hyphema also occurred in 25% of eyes after trabeculectomy in the intravitreal group. Sugimoto et al. (30) suggested that intravitreal injection simply decreases the neovascularization on the surface of the iris but did not entirely remove neovascularization in the iris stroma. However, the regression rate of NVI was much higher in the intracameral group, and no eyes experienced hyphema after trabeculectomy. This was possible because the NVI in iris stroma was likely eliminated but still needs validation from the histopathological examination of the resected iris.

A short-term clinical study described that patients with NVG exhibited a significant reduction in VEGF concentration in the aqueous humor 2 weeks after receiving an intracameral injection of bevacizumab, while this period is the crucial time of filtering bleb wound healing (31, 32). Because of the comprehensive anti-angiogenic effect of conbercept and its higher affinity to bind with VEGF than bevacizumab, we think the effective time should be longer than 2 weeks. During this process, the aqueous humor containing conbercept flows out through the filtering pathway, which would prevent angiogenesis and fibrosis in bleb wound healing and ultimately depress the scar formation of the filtering pathway. Although there was no significant difference in the incidence of malfunction filtering bleb between the two injection routes, we still think patients in stage III can benefit from intracameral injection to achieve a high concentration of the anti-VEGF agent in the aqueous humor.

For the patients in stage III, the anterior chamber angle had already been extensively closed, and the peripheral anterior synechia (PAS) could not be reversed. We found that the IOP remained at a high level after intravitreal injection and started to increase on the second day after intracameral injection. Based on this time window, we suggest performing trabeculectomy with MMC within 2 days after intracameral injection.

**Figure 2:**
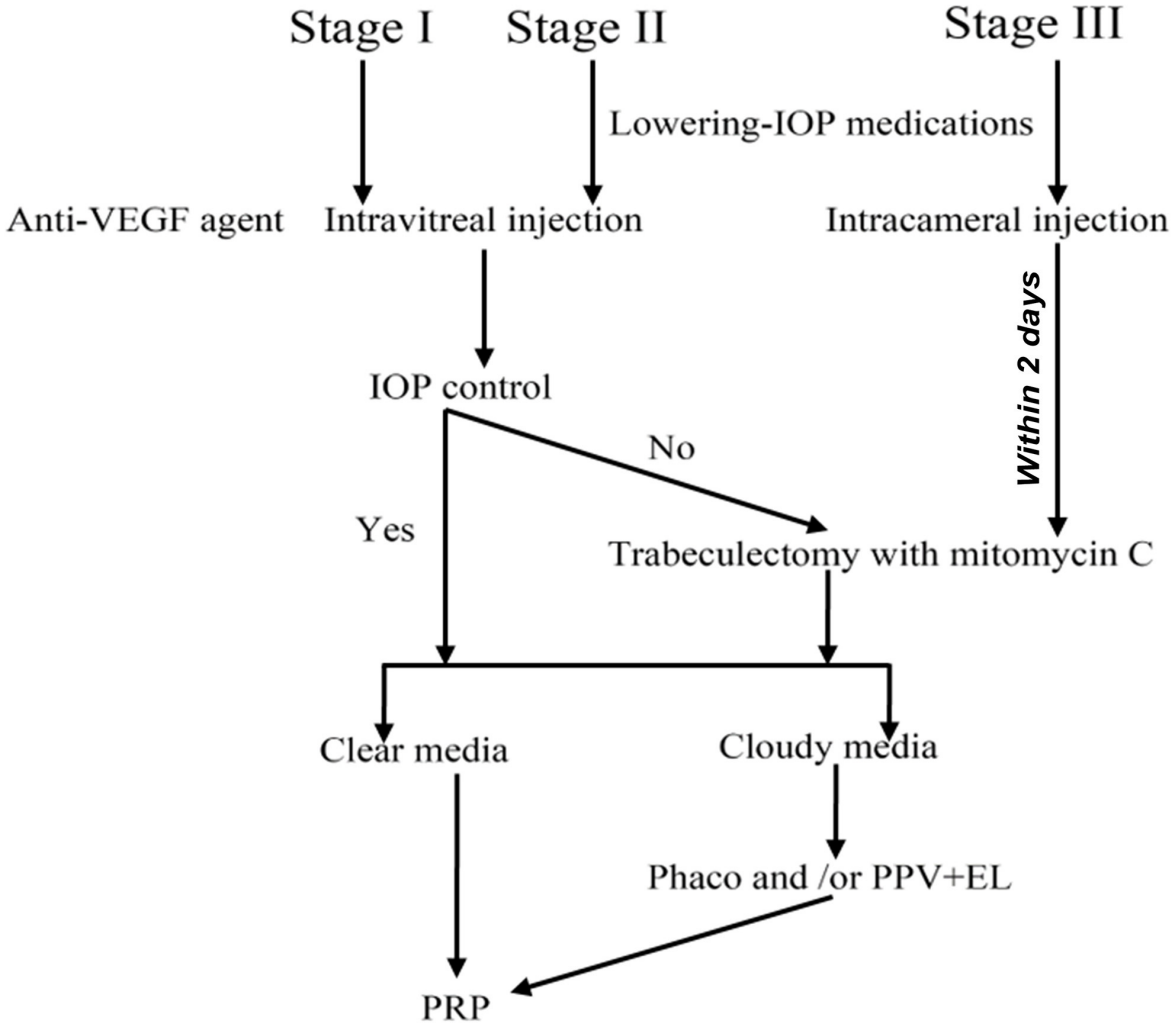
The optimized therapeutic regimen for neovascular glaucoma. Phaco, Phacoemulsification; PPV, Pars plana vitrectomy; EL, Endolaser; PRP, Pan-retinal photocoagulation.

Based on our research results, we proposed a therapeutic regimen for different stages of NVG, as shown in Figure 2. As for patients in stages I and II with good IOP control, starts from intravitreal anti-VEGF agent injection and finally complete PRP. For patients in stage III, intracameral instead of Intravitreal injection followed by trabeculectomy within 2 days, and PRP. Also, limitations of this study were a single-institution study, better to expand the sample size and extend follow-up time, etc.

### Typical Case

A 55-year-old man with a history of blurred vision for 5 months, and pain for 1 month in the right eye. Five months previously, the patient suffered blurry vision in the right eye and had FFA to diagnose CRVO at another hospital without receiving any treatment. One month ago, the patient had pain in the right eye and received three topical hypotensive eyedrops to control IOP. However, the patient gradually began to experience severe pain and finally came to our department. The ophthalmic examination showed that the visual acuity in the right eye was LP, the IOP was 48 mmHg, the cornea had edema, the pupil was 5 mm with slow light reflex, the iris had pronounced NVI, and the angle was closed more than 270° with obvious NVA and bleeding. Under maximal medication, the IOP was still 43 mmHg.

**Figure 3:**
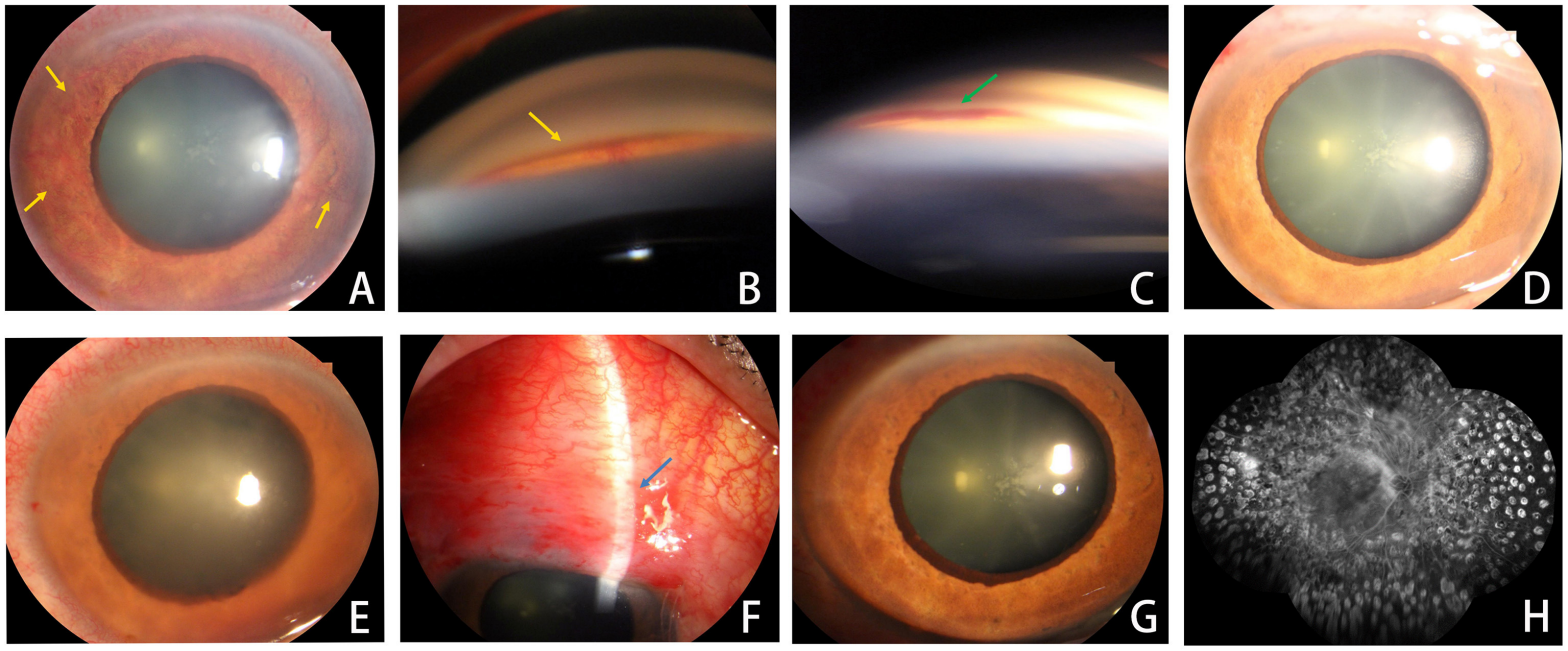
Ophthalmic examination of a typical case. **(A–C)** Before treatment, yellow arrows indicate NVI and NVA, a green arrow indicates bleeding at the angle; **(D)** 1 day after conbercept injection; **(E)** 2 days after conbercept injection; **(F)** 3 days after trabeculectomy with mitomycin C, blue arrow indicates filtering bleb; **(G)** 6 months after conbercept injection; **(H)** the completion of extra-PRP. PRP, Pan-retinal photocoagulation; NVI, neovascularization of Iris; NVA, neo-vessel at the anterior chamber angle.

Regarding the edema in the cornea, the fundus was not clear. At this time, the patient was diagnosed as “NVG (III), CRVO” and enrolled in our research. Initially, 0.5 mg conbercept was injected into the anterior chamber. One day later, the cornea was clear, the IOP was 17 mmHg, and the NVI had disappeared completely. However, while NVI had not recurred on the second day, the IOP had increased to 30 mmHg, and the cornea exhibited slight edema. Trabeculectomy with mitomycin C was performed immediately on this day, and no perioperative hyphema occurred. Three days later, the patient had diffused blebs, clear cornea, an IOP of 10 mmHg, and there was no recurrence of NVI. FFA showed ischemic CRVO. PRP was then completed promptly. Six months later, the visual acuity of the patient was 0.04, the IOP was 15 mmHg, the cornea was clear, and neo-vessels could not be found either on the iris or in the angle (Figure 3).

## Data Availability Statement

The raw data supporting the conclusions of this article will be made available by the authors, without undue reservation.

## Ethics Statement

The studies involving human participants were reviewed and approved by the Medical Ethics Board of the Second Affiliated Hospital of Xi'an Jiaotong University. The patients/participants provided their written informed consent to participate in this study. Written informed consent was obtained from the individual(s) for the publication of any potentially identifiable images or data included in this article.

## Author Contributions

SZ and FW designed the study. LB and YZ carried out the surgery. YW, WW, NH, and FT carried out the measurements and follow-ups. LB and XL wrote the manuscript. FW, SZ, LB, and XL were involved in the analysis of the results and further revision of the manuscript. All authors have read and approved the manuscript.

## Funding

This work was supported by the Research and development program of Shaanxi Province (no. 2021SF-161) and the Medical research project of Xi'an Science and Technology Action Plan [2019114613YX001SF041(1)]. The funders had no role in the study design, data collection, analysis, or manuscript writing.

## Conflict of Interest

The authors declare that the research was conducted in the absence of any commercial or financial relationships that could be construed as a potential conflict of interest.

## Publisher's Note

All claims expressed in this article are solely those of the authors and do not necessarily represent those of their affiliated organizations, or those of the publisher, the editors and the reviewers. Any product that may be evaluated in this article, or claim that may be made by its manufacturer, is not guaranteed or endorsed by the publisher.

## Abbreviations

NVG: Neovascular glaucoma
IOP: intraocular pressure
CPC: cyclophotocoagulation
NVI/NVA: neo-vessel at the iris or anterior chamber angle
ICRVO: ischemic central retinal vein occlusion
PDR: proliferative diabetic retinopathy
OIS: ocular Ischemic syndrome
VEGF: vascular endothelial growth factor
PIGF: Placental growth factor
wAMD: neovascular age-related macular degeneration (wet-AMD)
BCVA: best-corrected visual acuity
PRP: pan-retinal photocoagulation
FFA: fundus fluorescein angiography
MMC: mitomycin C.
